# An Investigation of the Psychometric Properties of the Chinese Trait Emotional Intelligence Questionnaire Short Form (Chinese TEIQue-SF)

**DOI:** 10.3389/fpsyg.2019.00435

**Published:** 2019-02-28

**Authors:** Anita Feher, Gonggu Yan, Donald H. Saklofske, Rachel A. Plouffe, Yan Gao

**Affiliations:** ^1^Department of Psychology, The University of Western Ontario, London, ON, Canada; ^2^Faculty of Psychology, Beijing Normal University, Beijing, China; ^3^School of Education, Beijing Normal University, Zhuhai, China

**Keywords:** trait emotional intelligence, TEIQue-SF, cross-cultural, confirmatory factor analysis, measurement invariance

## Abstract

The present study examined the psychometric properties of the Chinese version of the Trait Emotional Intelligence Questionnaire Short Form (TEIQue-SF). Analyses were performed using a sample of undergraduates (*N* = 585) recruited from four universities across China. Confirmatory factor analysis of the Chinese TEIQue-SF supported the one-factor structure of trait emotional intelligence. Measurement invariance analyses were conducted across the Chinese sample and a sample of Canadian undergraduate students (*N* = 638). Although the two samples demonstrated configural and partial metric invariance, scalar invariance was not found. Cross-cultural implications and explanations of the present findings, as well as suggestions for future research are discussed.

## Introduction

Emotional intelligence (EI) has garnered considerable research interest since its introduction to the research literature by Salovey and Mayer (1990). EI can be broadly conceived of as an individual differences variable describing emotion related distinctions (Petrides et al., 2007a; Petrides et al., 2018). One commonly-cited description of EI, referred to as trait EI (also called trait emotional self-efficacy), defines it as a cluster of behavioral dispositions and self-perceptions related to one’s emotions, positioned at the lower levels of personality hierarchies (Petrides and Furnham, 2001; Petrides et al., 2007b, 2016; Petrides, 2011). Trait EI is evaluated using self-report questionnaires (Petrides and Furnham, 2001), and a variety of psychometric measures have been developed to assess it (e.g., Bar-On, 1997; Schutte et al., 1998). One prominent measure is the Trait Emotional Intelligence Questionnaire (TEIQue; Petrides, 2009), which is the focus of the present study.

The TEIQue and its short form, the Trait Emotional Intelligence Questionnaire-Short Form (TEIQue-SF), were constructed to adequately cover trait EI’s sampling domain in line with trait EI theory (Pérez et al., 2005; Petrides, 2009, 2011). It operationalizes EI in accordance with the subjective nature and reporting of emotional experiences, and as a personality trait (Petrides et al., 2007b; Petrides, 2009, 2011). The measure comprises 15 facets which form four correlated factors, and can be further grouped to produce a global trait EI score (Petrides, 2009). Petrides (2009) defines these four factors as: *Well-Being* (well-being related feelings across time based around achievements, self-regard, and expectations), *Self-Control* (regulating and having control over emotions, impulses, and stress), *Emotionality* (ability to perceive, express, and connect with emotions in self and others, which can be used in creating successful interpersonal relationships), and *Sociability* (being socially assertive and aware, managing others’ emotions, and effectiveness in communication and participation in social situations).

Studies in Western countries utilizing the TEIQue have found numerous positive associations with global trait EI amongst undergraduate students, including higher resiliency (e.g., Vesely et al., 2014), greater proclivity to use adaptive coping strategies (e.g., Mikolajczak et al., 2008), and a positive relationship with academic performance (e.g., Sanchez-Ruiz et al., 2013; Petrides et al., 2018).

### Cross-Cultural Differences in Trait Emotional Intelligence

Cultures vary on multiple dimensions, including individualistic versus collectivistic societies (Hofstede, 1980, 2001; Triandis, 1995). Individualistic societies (e.g., North American) tend toward looser connections between people with more personal independence from collectives, and people are more influenced by personal motivations and goals (Hofstede, 1980; Triandis, 1995; Suh et al., 1998; Hofstede et al., 2010). Collectivistic societies (e.g., China) are characterized by tighter connections between people and strong identification with one or more collective in-groups, and valuing and prioritizing the norms and goals of these groups often over personal goals (Hofstede, 1980; Triandis, 1995; Suh et al., 1998; Hofstede et al., 2010).

Evidence suggests that emotions and latent personality traits manifest differently across cultural environments. For example, in line with collectivistic values of preserving interpersonal harmony, Siu and Chang (2011) reported that a Chinese sample was likely to control feelings of stress related to close relationships using avoidance or detachment. Cross-cultural differences have been found in norms surrounding expression of emotions, called display rules (Ekman and Friesen, 1969; Caruso, 2008). Matsumoto (1990) found that negative emotional displays among ingroup members and positive emotions toward outgroups were considered more appropriate in an American sample compared to a Japanese sample. Cross-cultural comparisons of personality traits have found positive associations between individualism and extraversion, with higher levels of extraversion amongst participants from individualistic compared to collectivistic countries (Furnham and Cheng, 1999; McCrae, 2001, 2002; Hofstede and McCrae, 2004).

Measures developed to assess trait EI tend to be factorially robust when assessed in different countries and cultures. The factor structure defined by trait EI measures other than the TEIQue show reasonable replicability across collectivistic countries such as China (Li et al., 2012; Kong, 2017), Japan (Fukuda et al., 2011) and Korea (Fukuda et al., 2012). Studies that examine the factor structure of the long version of the TEIQue have generally found that the factor structure is replicated (apart from some minor deviations) in other countries (e.g., Mikolajczak et al., 2007; Freudenthaler et al., 2008; Martskvishvili et al., 2013; Aluja et al., 2016). Studies examining the long version of the TEIQue in Chinese samples demonstrate partial replication of the factors. Mavroveli and Siu (2012) found a three-factor solution for the adolescent TEIQue, with sociability and emotionality combined to form a single factor. Gökçen et al. (2014) found a four-factor solution for the TEIQue. However, some of the facets did not load onto factors as expected.

There is mixed evidence in the research literature regarding cross-cultural comparisons of trait EI levels in more individualistic versus collectivistic countries (e.g., LaPalme et al., 2016). For example, Gökçen et al. (2014) found that British participants scored higher on global trait EI and on the four factors associated with the TEIQue compared to Chinese participants. Studies using other measures of trait EI have similarly found participants from a more individualistic country obtaining higher trait EI scores (Koydemir et al., 2013; Nozaki and Koyasu, 2016). However, another study using the TEIQue-SF found that the more collectivistic Cape Verdeans scored higher on global trait EI compared to Portuguese participants (Wilks et al., 2015). Using a different trait EI measure, a study comparing United States and Taiwanese academic leaders showed no significant differences in total EI, though some differences existed on subcomponent measures (Tang et al., 2010).

### Present Study

Trait EI was largely defined and developed in Western contexts, thus raising a need to examine the construct within non-Western samples (e.g., Gangopadhyay and Mandal, 2008). The present study aims to do so by examining the factor structure of the frequently-used trait EI measure, the TEIQue-SF, in a sample of Chinese undergraduate students. Although the long-form version of the TEIQue has been previously examined in a Chinese sample (e.g., Gökçen et al., 2014), to our knowledge, no previous study has undertaken this task using the short-form version of a Chinese-translated TEIQue. Short-form scales make important contributions and provide practical benefits to psychological research. For example, they prevent participant disengagement and are useful for studies including several questionnaires or repeated applications of the same questionnaire over multiple sessions (Austin et al., 2018). In line with findings of good model fit of the TEIQue-SF factor structure in a Spanish sample (Laborde et al., 2016) and factor analytic findings for the long version of the TEIQue in a Chinese sample (e.g., Gökçen et al., 2014), we expect to find good model fit for the factor structure using a Chinese translation of the TEIQue-SF.

Cultural variations in trait EI will also be explored by assessing the cross-cultural replicability of the TEIQue-SF. Cross-cultural measurement invariance will be used to compare the more collectivistic Chinese sample with a more individualistic Canadian sample. Measurement invariance is a means of assessing the psychometric equivalence of a construct (i.e., trait EI as assessed by the TEIQue-SF) across different groups (Putnick and Bornstein, 2016). When a construct is invariant across groups, it indicates that the different groups are attributing the same meaning to that construct (Putnick and Bornstein, 2016). Establishing cross-cultural invariance is important for comparisons across cultures on some construct (Mullen, 1995; Libbrecht et al., 2014; LaPalme et al., 2016). While factorial equivalence can be demonstrated within multiple cultures, it does not ensure measurement invariance of the measured construct across cultures (Byrne and Campbell, 1999; Byrne and Watkins, 2003). Therefore, a separate examination of measurement invariance of the TEIQue-SF is warranted. While several studies have examined the measurement invariance of other trait EI measures (e.g., Li et al., 2012), to our knowledge, no study has previously examined the cultural invariance of the TEIQue-SF in a Chinese and Canadian sample.

One comparative study using the long version of the TEIQue found that more individualistic participants scored higher on global and factor measures of trait EI (Gökçen et al., 2014). However, other studies making comparisons using participants from different countries or using different trait EI measures have found divergent results (e.g., Wilks et al., 2015). Based on inconsistencies in previous findings, the present study makes no specific hypotheses regarding individualistic-collectivistic group comparisons using the TEIQue-SF.

## Materials and Methods

### Participants

The present study included samples of Chinese and Canadian undergraduate university students. The Chinese sample (*N* = 585, 89 men, 447 women, and 49 unreported) was recruited from four Chinese universities. Their ages ranged from 16 to 26 years (*M* = 19.53, *SD* = 1.01). The Canadian sample (*N* = 638, 181 males and 456 females, 1 unreported) were recruited from a large Canadian university. Their ages ranged from 17 to 43 years (*M* = 18.50; *SD* = 2.14).

The present study followed the ethical guidelines required by the Canadian and Chinese universities, respectively. Written informed consent was given by participants in the Chinese sample, and explicit informed consent was given by the Canadian subjects using an online format prior to proceeding to questionnaires. All subjects gave informed consent in accordance with the Declaration of Helsinki. There were no formal ethics board requirements at the Chinese universities for survey method studies, and rather this was handled internally within the department. Ethics approval for the Chinese sample was therefore not required as per the Beijing Normal University’s guidelines and national regulations. With respect to the Canadian sample, ethical approval was given by the non-medical Western’s Research Ethics Board at the University of Western Ontario.

### Measures

For the Chinese sample, the trait EI data were obtained from a larger resiliency study (Wilson et al., 2018). The trait EI data for the Canadian sample was drawn from a larger personality study (Plouffe et al., in press). The trait EI measure has not been previously examined for either of these samples.

#### Trait Emotional Intelligence

The Trait Emotional Intelligence Questionnaire-Short Form (TEIQue-SF; Petrides, 2009) is a 30-item measure that evaluates global trait EI, though it can also be used to assess the four trait EI factors: Well-Being, Self-Control, Emotionality, and Sociability. As indicated by the TEIQue-SF scoring key, obtained from Petrides’ university laboratory website, items 3, 14, 18, and 29 only contribute to global trait EI, and not to any of the four factors. Therefore, these items were only used to calculate global trait EI scores. Participants responded to items using a 7-point Likert scale ranging from 1(*completely disagree* or *strongly disagree*) to 7(*completely agree* or *strongly agree*).

For the Chinese sample, the TEIQue-SF was translated into Mandarin following the recommended steps by Hambleton and Lee (2013). Care was taken to maintain content and lexical equivalence. The translation was done by faculty and graduate students who were native Chinese speakers as well as being highly fluent in English. Back translations were performed to ensure equivalence of the meaning for each item. Specifically, two psychology graduate students independently translated the English version into Chinese, and the two translations were examined for differences. Any differences were discussed, and final decisions for the scale were made by one of the Chinese authors of this paper. The Chinese version of the TEIQue-SF was then translated back into English with the aid of a professor at a Chinese university who has taught English for over 30 years. The translation process confirmed proper translation of the TEIQue-SF into Mandarin, allowing for distribution of the TEIQue-SF to Mandarin-speaking participants. The Canadian participants completed the English version of the TEIQue-SF as published by Petrides (2009).

### Data Analytic Strategy

The goal of the present study was twofold. The primary aim was to assess the factor structure of the TEIQue-SF in the Chinese sample, and the secondary aim was to evaluate cultural variations of the TEIQue-SF using cultural measurement invariance and mean comparisons. The factor structure for the TEIQue-SF used by Laborde et al. (2016) and Merino-Tejedor et al. (2018) was utilized in the present study. A one-factor model with the four trait EI factors (represented as indicators) loading onto global trait EI was tested using confirmatory factor analysis (CFA) in Mplus Version 7.4 (Muthén and Muthén, 1998-2015). When evaluating model fit, Root Mean Square Error of Approximation (RMSEA) values of 0.05 or below were considered good fit, values between 0.05 and 0.08 acceptable fit, and values between 0.08 and 0.10 were considered indicative of mediocre fit (Browne and Cudeck, 1993; MacCallum et al., 1996; Hu and Bentler, 1998). In line with Hu and Bentler’s (1999) suggestions, cut-off values close to 0.95 were considered demonstrative of good fit concerning Comparative Fit Index (CFI) and Tucker Lewis Index (TLI) values, and values below 0.08 were considered good fit regarding Standardized Root Mean Square Residuals (SRMR).

To assess cultural invariance of the TEIQue-SF, a series of CFA models were tested in hierarchal order using maximum likelihood estimation in Mplus Version 7.4 (Muthén and Muthén, 1998-2015). Configural invariance was assessed first in order to determine whether the basic organization of trait EI assessed with the TEIQue-SF (i.e., four trait EI factors well-being, self-control, emotionality, and sociability represented as indicators loading onto a global trait EI latent factor) is found in both cultures (Putnick and Bornstein, 2016). Metric invariance was assessed next to ascertain whether there is equivalence of factor loadings (i.e., equivalence in how the four trait EI factors load onto the global latent trait EI factor) in both cultural groups. Finally, scalar invariance was investigated to determine whether there is equivalence of intercepts. Scalar invariance establishes the connection between observed and latent score findings, such that equal values on latent trait EI will result in the same values on the observed trait EI factors for both Canadian and Chinese individuals (see Milfont and Fischer, 2010). If scalar invariance is not found, a comparison of latent mean scores between groups may not be meaningful. For example, although the two countries might differ on the observed Sociability factor, this may not be meaningfully associated with differences between countries in levels of latent trait EI if scalar invariance is not satisfied (Putnick and Bornstein, 2016). To compare the configural, metric and scalar invariance models, χ^2^ (at *p* = 0.01 significance level), CFI, and RMSEA difference tests were utilized. For these tests, ΔCFI values less than or equal to 0.01 in size, and ΔRMSEA values less than 0.015 in size, were utilized to indicate invariant models (Cheung and Rensvold, 2002; Chen, 2007).

## Results

### Descriptive Statistics and Bivariate Correlations

Descriptive statistics, Cronbach’s alphas, and bivariate correlations for the TEIQue-SF factor scores and total scores are presented for both the Chinese and Canadian data in Table 1. Means for both the Chinese and Canadian groups are similar to those found in previous studies (e.g., Herodotou et al., 2011; Laborde et al., 2016). Across both samples, the alpha coefficient for global trait EI was large (α = 0.88). However, the values for the trait EI factors ranged from small (α = 0.47) to large (α = 0.82) in the Chinese sample, and from acceptable (α = 0.67) to large (α = 0.85) in the Canadian sample. For both samples, the correlations between the factor and global TEIQue-SF scores were significant and positively related.

**Table 1:** Descriptive Statistics, Coefficient Alphas, and Correlations for Chinese and English Versions of the TEIQue-SF.

Variable	Mean	SD	α	1	2	3	4	5
**Chinese Sample**								
1 Global trait EI	4.73	0.64	0.88	1.00				
2 Well-being	5.10	0.96	0.82	0.83^∗^	1.00			
3 Self-control	4.53	0.80	0.65	0.81^∗^	0.64^∗^	1.00		
4 Emotionality	4.87	0.74	0.65	0.80^∗^	0.55^∗^	0.52^∗^	1.00	
5 Sociability	4.32	0.68	0.47	0.70^∗^	0.42^∗^	0.44^∗^	0.48^∗^	1.00
**Canadian Sample**								
1 Global trait EI	4.73	0.69	0.88	1.00				
2 Well-being	5.17	1.04	0.85	0.84^∗^	1.00			
3 Self-control	4.19	0.91	0.67	0.68^∗^	0.50^∗^	1.00		
4 Emotionality	4.79	0.85	0.67	0.73^∗^	0.48^∗^	0.28^∗^	1.00	
5 Sociability	4.77	0.87	0.71	0.70^∗^	0.48^∗^	0.32^∗^	0.40^∗^	1.00


*Listwise deletion used in correlation analysis for Chinese (*N* = 548) and Canadian (*N* = 633) data. ^∗^*p* < 0.01.*

### Confirmatory Factor Analysis of the Chinese TEIQue-SF

**FIGURE 1:**
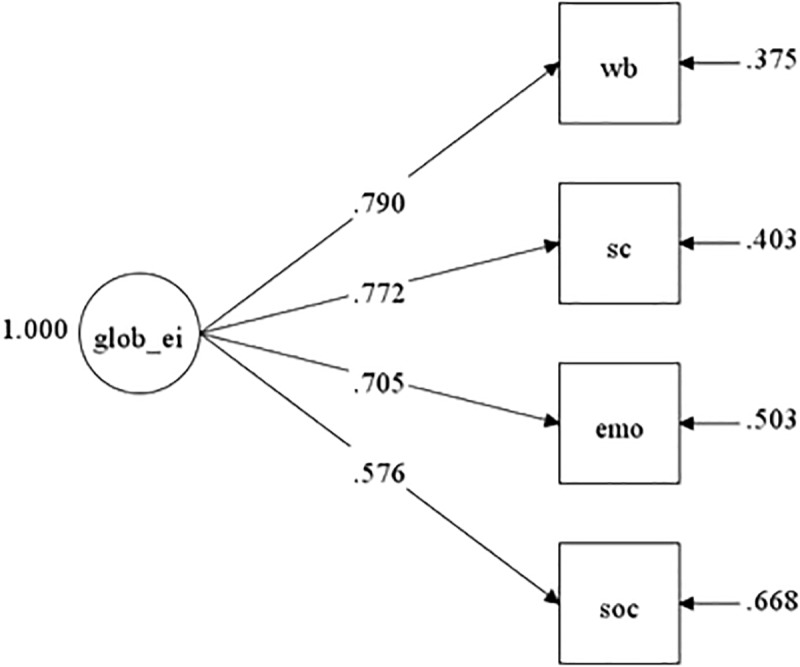
Confirmatory factor analysis of the Chinese TEIQue-SF (*N* = 554). glob_ei, global trait EI; wb, well-being; sc, self-control; emo, emotionality; soc, sociability.

A model with the four trait EI indicators (i.e., Well-Being, Self-Control, Emotionality, Sociability) loading onto one global trait EI factor was tested in the present study using CFA with maximum likelihood robust estimation. When all fit indices were considered, the fit for the one-factor model was acceptable: χ^2^_(2)_ = 12.188, RMSEA = 0.096 (90% CI = 0.049–0.151), CFI = 0.980, TLI = 0.939, SRMR = 0.024. The standardized factor loadings were generally strong, ranging from 0.58 to 0.79 (see Figure 1).

### Measurement Invariance Across Cultures

To make cross-cultural comparisons and establish the generalizability of the TEIQue-SF, invariance of the factor structure of the TEIQue-SF must be established (Reise et al., 2000; Widaman and Reise, 1997) Therefore, nested CFA models were compared using both the Chinese and Canadian samples to determine whether measurement invariance of the TEIQue-SF was found across cultures (see Table 2). According to the CFI index, the configural model demonstrated acceptable fit χ^2^_(4)_ = 28.24; *p* < 0.001, RMSEA = 0.100 (90% confidence interval [CI] = 0.067–0.137), CFI = 0.981. Examination of the chi-squared change index showed that there was a significant difference between the metric model with constrained factor loadings and the configural model, Δχ^2^_(3)_ = 19.08, *p* < 0.01, though large sample sizes may have been responsible for this influence (Cheung and Rensvold, 2002). Although examinations of RMSEA change index showed non-significant differences between the models, ΔRMSEA = -0.002, the CFI change index also showed significant differences between the models, ΔCFI = -0.013. To test for partial metric invariance, an examination of the modification indices led to the decision to free the self-control factor. Subsequent chi square, RMSEA, and CFI difference tests were non-significant, providing support for partial metric invariance, Δχ^2^_(2)_ = 7.80, ΔRMSEA = -0.009, and ΔCFI = -0.005. Scalar invariance was tested only on metric-invariant loadings; therefore, Self-Control was left free to vary (Putnick and Bornstein, 2016). Findings of chi square, RMSEA, and CFI difference tests did not support scalar invariance across cultural groups, Δχ^2^_(2)_ = 125.82, *p* < 0.01, ΔRMSEA = 0.088, and ΔCFI = -0.099, which inhibits meaningful assessment of latent mean differences across cultural groups, and overall testing of group differences using the TEIQue-SF (Putnick and Bornstein, 2016).

**Table 2:** Cultural Measurement Invariance Fit Indices.

Model	χ^2^ *(df)*	RMSEA	RMSEA 90% C.I.	CFI
1. Configural invariance	28.236^∗^(4)	0.100	0.067–0.137	0.981
2. Metric invariance	47.314^∗^(7)	0.098	0.072–0.125	0.968
3. Partial metric invariance	36.036^∗^ (6)	0.091	0.064–0.121	0.976
4. Partial scalar invariance	161.856^∗^(8)	0.179	0.155–0.203	0.877


*^∗^*p* < 0.001.*

A lack of measurement invariance indicates that trait scores on the TEIQue-SF are not comparable across the Chinese and Canadian samples assessed in the present study (Reise et al., 1993). Therefore, mean differences on this measure or correlations using this measure can be potentially misleading (Reise et al., 1993). Therefore, further analyses to investigate group mean differences on the TEIQue-SF between the two groups were not performed.

## Discussion

Overall, the present study had two main purposes: (1) to determine whether the factor structure of the TEIQue-SF (Petrides, 2009) was upheld in a non-Western context, and (2) to evaluate whether cross-cultural differences exist in the conceptual interpretation of the TEIQue-SF. Results of the present study demonstrated that while the factor structure of the TEIQue-SF was replicated, there were differences in the meaning of latent trait EI when assessed in a more collectivistic context in comparison to a more individualistic sample.

The present study examined the robustness of the factor structure of the TEIQue-SF (Petrides, 2009) in a Chinese undergraduate sample. Trait EI has been primarily defined in a Western context, therefore requiring the need for the construct to be psychometrically validated in non-Western samples (e.g., Gangopadhyay and Mandal, 2008). This study aimed to do so by examining the fit of a one-factor model (i.e., global trait EI and Petrides’ four trait EI factors) using the TEIQue-SF.

Results of the CFA demonstrated that the Mandarin translation of the TEIQue-SF had acceptable fit. Previous studies have cross-culturally replicated the factor structure of the TEIQue (e.g., Freudenthaler et al., 2008). While there are fewer studies similarly examining the factor structure of the TEIQue-SF, Laborde et al. (2016) and Merino-Tejedor et al. (2018) found evidence of good model fit for the TEIQue-SF in their Spanish samples. Additional studies that have investigated the factor structure of the TEIQue-SF have chosen to include items or facets in their CFA models (e.g., Jacobs et al., 2015; Snowden et al., 2015). The current study did not, following on Petrides (2009) suggestion that the TEIQue-SF was not designed to be factor analyzed at the item level or scored at the facet level.

Internal reliability analysis of the TEIQue-SF in the Chinese sample demonstrated high alpha scores on global trait EI, but scores were lower when assessed at the TEIQue factor level. While internal consistencies for factor scores on the short form are expected to be slightly lower (Petrides, 2009), the Sociability factor had lower than expected alpha values in the current study. Some other studies have also reported low alpha coefficients for Sociability on the TEIQue-SF (e.g., Petrides et al., 2010).

Findings that a measure like the TEIQue-SF has a similar factor structure within different cultural contexts do not guarantee that the measure will perform equivalently across cultures (Byrne and Campbell, 1999). Analysis of cultural invariance demonstrated that while configural and partial metric invariance for the TEIQue-SF were achieved, scalar invariance was not achieved across cultural groups. Therefore, the trait EI measure performs differently and has different meanings for Chinese participants in comparison to Canadian participants. Such a finding is important for moving forward with cross-cultural comparisons using this measure, and inhibits meaningful interpretation of trait EI comparisons in the present study.

One potential explanation for lack of invariance is measurement bias (Hong et al., 2003). Misunderstandings of items in different cultures or potential translational issues might serve as potential explanations. Previous studies have also reported cultural factors having an influence on responses to questionnaires, which may affect invariance. For example, analysis of multiple countries revealed that individualism was negatively related to a middle response style and acquiescent responding (Harzing, 2006). Other studies have also found evidence for individuals from more collectivistic countries being more likely to endorse midpoint values, and having less extreme scores on scales compared to individualists (Chen et al., 1995; Takahashi et al., 2002). However, an examination of the data regarding means and standard deviations from both countries suggests these latter points regarding response style are not particularly applicable to the current study.

Differences across cultures in whether and/or to what extent emotionality, well-being, self-control, and sociability define latent trait EI across cultures may also serve as a potential explanation for findings of non-invariance. For example, self-control factor loadings were not equivalent across Chinese and Canadian samples in the present study. A possible explanation may be found in greater value ascribed to control over one’s inner emotions and desires amongst more collectivistic individuals (Markus and Kitayama, 1991). In the development of their Asian American Values Scale, Kim et al. (2005) listed emotional self-control as a central value. This potentially suggests that self-control might be more strongly related to Chinese participants’ self-perceptions of emotion-related competencies. Findings from the present study demonstrate a need for further exploration of how cultural differences account for different interpretations of trait EI.

### Limitations and Future Directions

Some limitations with the present study should be considered and used to guide future research. The present study’s use of undergraduate participants made it possible to compare the Chinese sample to a demographically similar Canadian sample. Future studies, however, should also compare the psychometric properties of the TEIQue-SF among participants of different age brackets and different groups within a country. The present study also used a predominantly female sample, and future studies should recruit participants with a more equal gender balance. Differences in trait EI levels across different university faculties have been previously observed (e.g., Sanchez-Ruiz et al., 2010) which might warrant further study. Future cross-cultural research should include measures of an individual’s individualism and collectivism to assess these relationships with trait EI, as well examinations of other cultural factors that might be driving these differences. Finally, our results showed that trait EI manifests differently across the two countries, and future research should examine reasons for this further.

## Author Contributions

All authors listed have made a substantial, direct and intellectual contribution to the work, and approved it for publication.

## Conflict of Interest Statement

The authors declare that the research was conducted in the absence of any commercial or financial relationships that could be construed as a potential conflict of interest.
